# Advancing Behavioral Intervention and Theory Development for Mobile Health: The HeartSteps II Protocol

**DOI:** 10.3390/ijerph19042267

**Published:** 2022-02-17

**Authors:** Donna Spruijt-Metz, Benjamin M. Marlin, Misha Pavel, Daniel E. Rivera, Eric Hekler, Steven De La Torre, Mohamed El Mistiri, Natalie M. Golaszweski, Cynthia Li, Rebecca Braga De Braganca, Karine Tung, Rachael Kha, Predrag Klasnja

**Affiliations:** 1 Center for Economic and Social Research, Department of Psychology, University of Southern California, Los Angeles, CA 90089, USA; 2 Manning College of Information and Computer Sciences, University of Massachusetts Amherst, Amherst, MA 01003, USA; marlin@cs.umass.edu (B.M.M.); karine@cs.umass.edu (K.T.); 3 Khoury College of Computer Sciences, Northeastern University, Boston, MA 02115, USA; m.pavel@neu.edu; 4 Bouve College of Health Sciences, Northeastern University, Boston, MA 02115, USA; 5 Control Systems Engineering Laboratory, School for Engineering of Matter, Transport, Energy, Arizona State University, Tempe, AZ 85287, USA; daniel.rivera@asu.edu (D.E.R.); melmisti@asu.edu (M.E.M.); rachael.kha@gmail.com (R.K.); 6 Herbert Wertheim School of Public Health and Human Longevity Science, University of California, San Diego, CA 92093, USA; ehekler@ucsd.edu (E.H.); ngolaszewski@health.ucsd.edu (N.M.G.); 7 Design Laboratory, University of California, San Diego, CA 92093, USA; 8 Center for Wireless and Population Health Systems, University of California, San Diego, CA 92093, USA; 9 Department of Population and Public Health Sciences, University of Southern California, Los Angeles, CA 90032, USA; sdelator@usc.edu (S.D.L.T.); licynthi@usc.edu (C.L.); rbraganc@usc.edu (R.B.D.B.); 10 The VA San Diego Healthcare System, San Diego, CA 92161, USA; 11 School of Information, University of Michigan, Ann Arbor, MI 48109, USA; klasnja@umich.edu

**Keywords:** Mobile Health, mHealth, digital health, intensive longitudinal data, physical activity, real-time interventions, new technologies, psychosocial theory, behavioral health

## Abstract

*Background:* Recent advances in mobile and wearable technologies have led to new forms of interventions, called “Just-in-Time Adaptive Interventions” (JITAI). JITAIs interact with the individual at the most appropriate time and provide the most appropriate support depending on the continuously acquired Intensive Longitudinal Data (ILD) on participant physiology, behavior, and contexts. These advances raise an important question: How do we model these data to better understand and intervene on health behaviors? The HeartSteps II study, described here, is a Micro-Randomized Trial (MRT) intended to advance both intervention development and theory-building enabled by the new generation of mobile and wearable technology. *Methods*: The study involves a year-long deployment of HeartSteps, a JITAI for physical activity and sedentary behavior, with 96 sedentary, overweight, but otherwise healthy adults. The central purpose is twofold: (1) to support the development of modeling approaches for operationalizing dynamic, mathematically rigorous theories of health behavior; and (2) to serve as a testbed for the development of learning algorithms that JITAIs can use to individualize intervention provision in real time at multiple timescales. *Discussion and Conclusions*: We outline an innovative modeling paradigm to model and use ILD in real- or near-time to individually tailor JITIAs.

## 1. Introduction

Unhealthy behaviors, including physical inactivity, unhealthy diets, smoking, inadequate sleep, and excessive alcohol use, contribute to chronic conditions which account for 86% of all healthcare spending in the U.S [1]. Despite decades of research, available behavior-change interventions remain only moderately effective short-term [2], largely lack effectiveness for sustaining long-term behavioral maintenance [3,4], and are often too costly to be scalable. A key reason for this low level of effectiveness is a mismatch between the current theories of behavior change on which most interventions continue to be based and the complexity of the processes they are trying to target. Many current interventions tend to be based on a relatively narrow range of health-behavior theories [5]. These theories conceptualize health behavior statically, in that they identify constructs that influence health behavior, but often lack specification of the dynamics of these relationships. The exact constructs a theory focuses on differ from theory to theory (e.g., social-cognitive theory, theory of planned action, health belief model, etc.), but their *form*—that they can be presented by box-and-arrow diagrams depicting time-invariant relationships among constructs—is remarkably consistent. This fact is in large part due to the health-behavior theorists’ reliance on temporally sparse cross-sectional data that have characterized applied behavioral science for much of its history [6].

In recent years, there has been a growing realization that the static relationships postulated by the health-behavior theories do not accurately capture the actual processes that underlie health behavior change [7]. Health behaviors—like human behavior more generally—are highly dynamic, and are shaped, from moment to moment, by myriad factors, including physical context, social norms and expectations, physiological responses like stress, and, yes, psychosocial constructs like expectancies or beliefs that are the bread and butter of classic theories of health behavior. The key point, though, is that these various influences are constantly changing and feasibly co-interacting with one another from one moment to the next [6]. For example, a person may go from their home, where unhealthy food is readily available, to the office, where it is not, come into contact with different people over the course of the day who may signal, explicitly or implicitly, different norms around eating or weigh, see a commercial advertising fast food, see the calorie values for different foods while ordering lunch, and look for plane tickets for a beach vacation a month away. Each of these events, and many more besides, could influence what, when, and how much the person decides to eat that day. Such time-varying, co-interacting influences are poorly accounted for by the classic health-behavior theories, and, as a result, interventions based on these theories have not been able to adequately manage the complexity and dynamics of real-life health behavior change.

Recent advances in mobile and wearable technologies have led to two developments that may allow the field of health behavior change to move beyond this status quo. First, they have enabled a new form of intervention, called “Just-in-Time Adaptive Intervention” (JITAI) [8]. The objective of JITAIs is to interact with the individual at the most appropriate time and provide the most appropriate support depending on the continuously acquired information about the participant’s physiology, behavior, and contexts. JITAIs, therefore, have the potential to continuously adapt to changing contexts and personalize intervention provision to individual needs and abilities in order to optimize support for behavior change over time.

Second, through passive sensing (e.g., GPS, activity, heart rate, galvanic skin response, etc.), in-the-moment self-report like ecological momentary assessment (EMA), and digital footprints (e.g., text-messaging logs, application-use data, etc.), mobile and wearable devices are enabling collection of rich, temporally-dense data sets about potential influences on health behavior, as well as granular data about how those behaviors themselves (e.g., physical activity) evolve over time. Unfortunately, data from sensors can have a great deal of missingness due to non-wear or technical problems. Self-report data are limited by participant burden considerations and non-adherence. Additionally, data about many constructs that are potentially crucial for understanding behavioral dynamics in detail, such as social interactions or psychological states like salience, are still incredibly difficult to obtain. Yet, even with these limitations, mobile and wearable technologies are enabling researchers to collect far more granular and multifaceted data about the time-varying influences on health behaviors than has ever been possible.

Availability of these advances raises an important question: How do we use these data to better understand and intervene on health behaviors? On the intervention side, the promise of JITAIs is contingent on the system’s identifying moments of need for specific types of support and moments when someone would have the opportunity to act and be receptive to support. In addition, JITAIs need to be able to evaluate the benefits of different types of support provided in different contexts in order to adapt intervention provision towards support that facilitates sustained movement towards desired states, such as consistently engaging in physical activity. JITAIs, therefore, need to identify idiographic patterns about whether and how different types of support will be helpful for each person, both in a given moment and over time. Facilitating learning of such patterns is a key challenge in JITAI development (It may seem that reinforcement-learning (RL) approaches offer a solution to the development of JITAIs that do not rely on behavioral theory, since they can learn patterns in responsiveness purely through data. However, the high noise and sparsity in mHealth data mean that JITAIs are limited in what they can learn through purely data-driven approaches. Thus, even RL-based JITAIs benefit from models of health behavior and behavior change, even only as a basis for the initial intervention-provision policy which can then be further adapted through data). On the theory-development side, we need ways to combine data collected across multiple time scales (minutes, hours, days, weeks, etc.), conceptualize how constructs of interest evolve over time based on different types of indicators (sensor data, repeated responses to EMA), etc.), and deal with the abundance of missingness found across the data streams. Put differently, JITAIs establish the need for not only testing if interventions and intervention components work, but also advance fundamental understanding of processes of behavior change as it manifests in real-world contexts [9,10]. In other words, we need to learn how to theorize dynamically in the messy world of health behavior.

The HeartSteps II study, described here, is intended to advance both intervention development and theory-building enabled by the new generation of mobile and wearable technology. The study involves a year-long deployment of HeartSteps, a JITAI for physical activity and sedentary behavior, with 96 sedentary, overweight, but otherwise healthy adults. The study goes beyond intervention testing, however. The central purpose is twofold: (1) to support the development of modeling approaches for operationalizing dynamic, mathematically-rigorous theories of health behavior; and (2) to serve as a testbed for the development of learning algorithms that JITAIs can use to individualize intervention provision in real time at multiple timescales.

This study builds on a set of NIH-funded micro-randomized trials (MRTs) [11,12,13], which were conducted to optimize HeartSteps and assess its just-in-time intervention components for impact on walking and decrease sedentary behavior in patients with cardiovascular disease. An MRT is an experimental design in which JITAI intervention components are repeatedly randomized for each participant over time to assess their impact on their intended proximal (i.e., near-term) outcomes, and to inform the design of JITAI decision rules, such as when and how much to intervene. MRTs often leverage densely sampled intensive longitudinal data from mobile and wearable devices as they require both frequent sampling of proximal outcomes as well as assessments of contextual and psychosocial factors, such as location, mood, or weather, which may influence an individual’s receptivity to an intervention and its effectiveness. With such data, MRTs are designed to gauge the effectiveness of micro-randomized intervention components on their proximal outcomes and assess how the effectiveness changes over time and across contexts.

Like the initial HeartSteps studies, the HeartSteps II study includes micro-randomization of several intervention components, but it also collects a range of other data intended to facilitate multi-timescale modeling to support JITAIs. In what follows, we describe the HeartSteps intervention, the protocol for its deployment in a year-long study with 60 overweight, sedentary adults, and we provide a brief overview of the modeling approaches that we are developing using the data collected in this study.

## 2. Materials and Methods

The HeartSteps II study protocol and consent procedures were approved by the University of Southern California Institutional Review Board before enrolling participants. The trial is registered on clinicaltrials.gov and the full study protocol and results will be published on clinicaltrials.gov (26 April 2021) upon completion of the trial.

### 2.1. Research Design

HeartSteps II is a single-arm, year-long MRT. Participants wear a Fitbit activity tracker but do not receive the HeartSteps intervention for a week-long baseline period. Following the baseline week, participants are then given access to the HeartSteps mobile-phone application (see Section 2.3 for details). For the next 12 months, they are expected to wear the Fitbit every day for at least 10 h per day, and to use the HeartSteps app on their phones to help them plan how they will be active, set activity goals, and to receive just-in-time support for engaging in short bouts of activity and disrupting sedentary behavior. These walking suggestion and anti-sedentary suggestions are continuously micro-randomized for each participant, as are the motivational messages that are sent as part of a survey that participants receive every morning. At the end of the study, participants are interviewed about their experience and are compensated for their participation.

### 2.2. Aims

The aims the HeartSteps II study are to: (1) refine and develop temporally dense and appropriately sampled measures that enable us to study dynamic processes of theoretical constructs that influence our target behaviors, (2) enhance HeartSteps with the measures developed, and collect a full year of data from a cohort of at least 60 sedentary, adults with overweight/obesity, (3) develop a modeling framework to operationalize dynamic and contextualized theories of behavior in an intervention setting, and (4) apply this framework to create dynamic models of intervention engagement and intervention response starting with constructs from the Self-Determination theory [14,15].

### 2.3. HeartSteps Intervention Components

HeartSteps is a JITAI for physical activity and sedentary behavior. From the user perspective, the intervention consists of three main components: the Fitbit Versa Lite activity tracker, the HeartSteps app that runs on participants’ smartphones, and a custom HeartSteps clock face that runs on the Fitbit. The rest of this section describes each of these components and the intervention content that they contain.

#### 2.3.1. Fitbit

The Fitbit Versa Lite is a wrist-worn, watch-style physical activity tracker. Activity data recorded by the Fitbit device is automatically pushed by the Fitbit server to the HeartSteps system using Fitbit’s subscription API, and it becomes visible inside the HeartSteps smartphone application. In addition to recording participants’ activity, Fitbit gives users always-visible feedback on the current step count and heart rate, and it provides them with positive reinforcement (in the form of a fireworks display) if they reach the daily step goal that is part of the Fitbit application (but is not explicitly used in HeartSteps).

#### 2.3.2. HeartSteps App

The HeartSteps application contains both “pull” components that are always available to participants inside the app, and “push” components that are provided as push notifications. The application is also used for assessments, which are partially integrated with intervention provision in order to make them less burdensome to participants.

**Figure 1:**
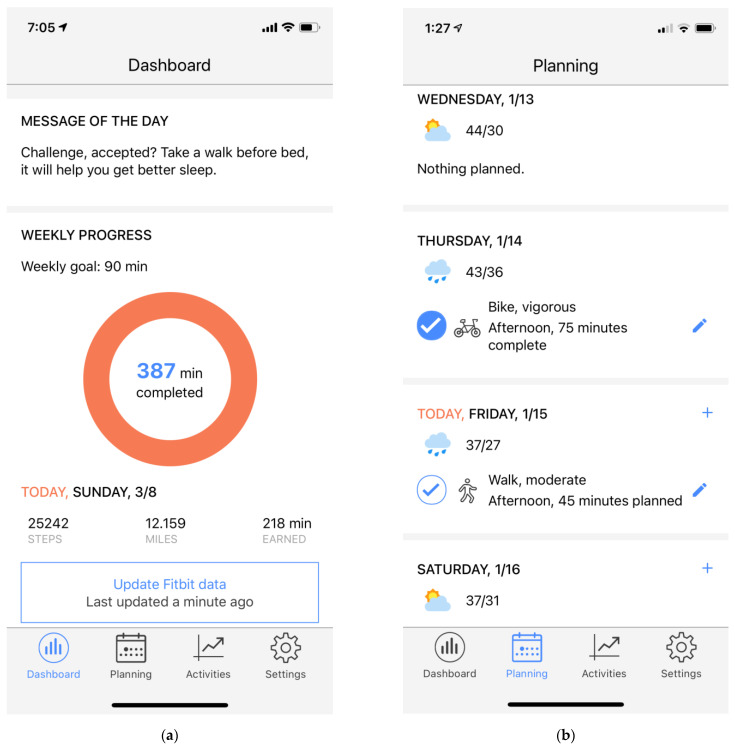
Screenshots of the HeartSteps application: (**a**) HeartSteps dashboard; (**b**) Main planning screen.

The HeartSteps app consists of the three *pull components*. These are all accessible through the tabs along the bottom of the application screen: (1) Dashboard: The dashboard is the home screen of the application, and is shown to users whenever they enter the application or complete interacting with one of HeartSteps notifications (Figure 1a). The dashboard implements three behavior-change techniques (BCTs) [16,17]: self-monitoring, feedback on goal progress, and reminders of activity plans. At the center of the dashboard is a circular display that shows the participant’s activity goal for the week (in minutes of MVPA) and current progress toward that goal. Below the progress display, the dashboard shows the current daily step count recorded by the Fitbit, and any activity plans that the participant made for the current day. (2) Planning: Through the planning tab (Figure 1b), participants are able to make plans for when they are going to be active during the current week. Planning is intended to implement the construct of implementation intentions [18,19], enabling participants to record on what day they will be active, what activity they plan to do, for how long, and during what part of the day. Research on implementation intentions has found this level of specificity has been shown to increase the likelihood that the plans will, in fact, be executed. (3) Activity log: Through the activity log, participants are able to see automatically-detected and manually-logged activities, create a new activity record (e.g., for activities that were not detected or if the participant did not wear the Fitbit), and see all-time statistics for their activity: hours of active time, total number of steps recorded since they started using HeartSteps, and the total distance traveled. These all-time statistics are intended to provide a longer time-frame feedback on what the participant is accomplishing through his or her efforts to be active.

**Figure 2:**
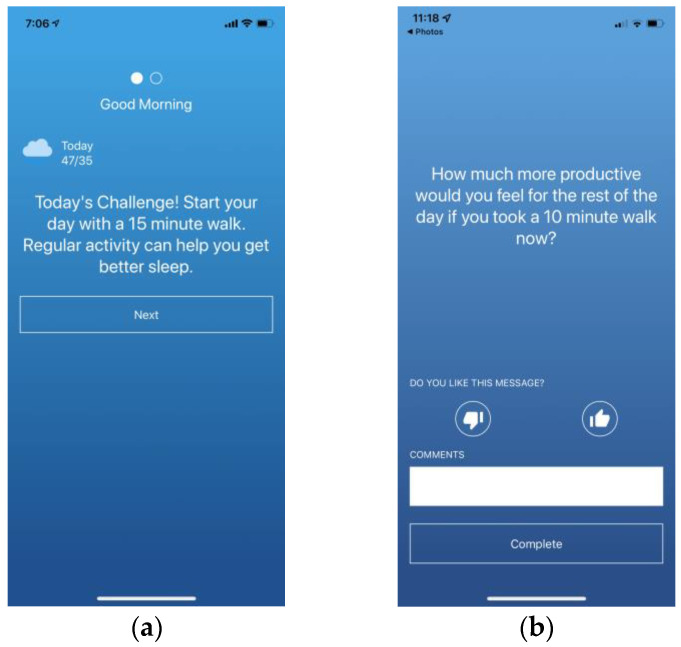
Screenshots of HeartSteps push notifications: (**a**) Morning motivational message; (**b**) walking suggestion.

In addition to these always-accessible features, HeartSteps includes several *push interventions* that are provided as push notifications. These include the following: (1) Morning motivational messages: Motivational messages (Figure 2a) are brief messages encouraging participants to be active that day using either a promotion or prevention framing (see the section on randomization below). They are delivered as part of the morning-survey interaction and are sent at 6 a.m. (when most phones are by default in Do Not Disturb mode) in order to be present on the participants’ phones when they wake up. When a motivational message is provided, a companion message with the same framing is displayed on the app dashboard to make the content salient during the day. (2) Walking suggestions: Walking suggestions (Figure 2b) are short messages, tailored to the participant’s current context (time of day, weekday/weekend, weather, and location [work, home, other]) intended to encourage participants to engage in short bouts of activity. Tailoring to the current context is intended to make the messages immediately actionable and thus allow them to act as cues to action [20,21]. Walking suggestions are randomized five times a day at user-specified times in the morning, mid-day, mid-afternoon, late-afternoon, and evening, using a reinforcement-learning algorithm, as described in Randomization section below. (3) Anti-sedentary suggestions: Anti-sedentary suggestions are intended to disrupt prolonged periods of inactivity by suggesting to participants to get up and briefly move around. Like walking suggestions, they are tailored to the time of day, weather, weekday/weekend, and location to make them actionable. They are randomized to be provided if a participant is currently wearing the Fitbit tracker and the tracker has detected fewer than 150 steps in the previous 40 min. Checking whether the participant is currently sedentary is done every five minutes by the HeartSteps clock face running on the participants’ Fitbit devices. (4) Weekly reflection: At the end of the week at a user-selected time (by default, 8 p.m. on Sundays), participants are prompted to engage in a brief weekly reflection. This interaction includes a short survey asking them about their experiences with trying to be active over the last week (see the section on Assessments below), to report any barriers to activity and think about whether those barriers will continue into the following week, to review and set an activity goal for the coming week, and, if they want to, to make plans for when they will be active next week in order to reach their activity goal, using the same type of planning interface that they can access through the app.

In addition to the pull and push components described above, a core intervention in HeartSteps is an adaptive weekly activity goal. The purpose of this goal is to gently shape participants to the level of activity recommended by the clinical guidelines [21], namely, 150 min of MVPA per week. The HeartSteps system sets this goal automatically at the end of each week for the following week based on the number of minutes of MVPA that participants achieved in the week that just ended. The algorithm for this is very simple: the next week’s goal is set to 20 min more than the participant accomplished the current week, unless this would result in more than 150 min, in which case the goal is set to the guidelines level of 150 min. In spite of its simplicity, this algorithm is able to quickly adjust to the changes in participants’ circumstances, since if a participant is going through a busy period or got sick and was less active for a week, the goal immediately adjusts to take account of this change.

Although the goal is adjusted automatically, during the weekly reflection participants are given an option to adjust it up or down, based on what they know about the week ahead. On the goal review screen, they are also asked about their self-efficacy for achieving this goal, which is intended to help them set realistic goals that they can, in fact, accomplish. Finally, although the system will never automatically set the goal to more than 150 min of MVPA per week, during the weekly review participants are able to manually adjust it to a number higher than that. If a participant does not do the weekly review (i.e., by ignoring or clearing the weekly-review notification) the system will still set the goal automatically, as described above, so the goal feedback on the dashboard continues to work properly.

#### 2.3.3. HeartSteps Clock Face

Fitbit Versa trackers allow developers to create custom clock faces. The HeartSteps system includes a custom clock face that participants are instructed to install when they first join the study. Like the default Fitbit clock face, the HeartSteps clock face provides real-time feedback on step count and heart rate. Its main purpose, however, is to provide HeartSteps with near-real-time step data that is used to assess if the participant has been sedentary for an extended period and may benefit from an anti-sedentary suggestion. Since these suggestions are intended to be provided only if the participant is currently sedentary, a functional HeartSteps clock face is a requirement for their provision as the Fitbit API does not provide activity updates often enough to allow for accurate intervention timing.

### 2.4. HearSteps Intervention Design: Randomization, Availability and Proximal Outcomes

The HeartSteps II study micro-randomizes three of the HeartSteps push intervention components: motivational messages, walking suggestions, and anti-sedentary suggestions. While the motivational messages are randomized using a standard fixed probability, both the walking and anti-sedentary suggestions are randomized using algorithms that personalize randomization probabilities to each participant. Randomization is performed as follows:

#### 2.4.1. Motivational Messages

Motivational messages are randomized for each participant each day of the study (following the baseline week). ach day, for each participant, the system randomizes whether to send a motivational message, and if so, which motivational framing [22,23] of the message to use. There are four framings of motivational messages: (1) prevention framing with focus on being active; (2) promotion framing with the focus on being active; (3) prevention framing with the focus on not being sedentary; and (4) promotion framing with the focus on not being sedentary. Motivational messages are randomized with the probability of 3/7 that no message is sent, and probability of 1/7 that a message is sent with each of the four framings listed above. In other words, some type of a motivational message is provided on a little more than half of the mornings (p = 4/7), while the rest of the time (p = 3/7) no motivational message is included in the morning survey.

Proximal outcome for motivational messages—the near-term outcome that the motivational messages are intended to impact—is the daily step count. We hypothesize that on days when participants receive motivational messages, they will have more steps than on days when they do not receive motivational messages.

#### 2.4.2. Walking Suggestions

Walking suggestions in HeartSteps are randomized using a contextual bandit reinforcement learning algorithm [13] that was developed based on the data from the first HeartSteps MRT [12]. Every night, the algorithm, a Bayesian Thompson sampler, analyzes the data about each participant’s responsiveness to previously sent walking suggestions and then adjusts the weights used to determine intervention provision probabilities for the next day. The goal of this update process is for the algorithm to understand the extent to which a participant is being responsive to walking suggestions and whether his/her responsiveness is different in different contexts (location, time of day, weather, etc.). Based on this information, the algorithm then adjusts future intervention provision. If a participant is responsive, the overall probability of providing walking suggestions in the future goes up, and if the responsiveness drops—say, due to habituation—intervention probabilities go down to give the individual a break. Similarly, if a participant is responsive in a particular context (e.g., in the morning, on the weekends, when they are at home), the probability of receiving a walking suggestion in that context will go up; if a participant is unresponsive in another context (e.g., mid-afternoon on weekdays while the participant is at work), that person’s probability of receiving a walking suggestion in that context will go down. The overall goal of this bandit algorithm is to adjust, over time, intervention provision for each person so that walking suggestions are provided in contexts and at frequencies the person is most likely to be responsive to, thus maximizing effectiveness for motivating near-term activity while minimizing participant burden. At each decision point (five times per day), the HeartSteps system saves the probability that was used to randomize a walking suggestion for each participant, so that the data about walking suggestions can be analyzed after the study is over.

The proximal outcome for walking suggestions is the 30-min step count following the decision time. We hypothesize that when participants are randomized to receive a walking suggestion, they will walk more in the 30 min following the provision of the suggestion than when they are randomized to not receive a walking suggestion.

Availability for walking suggestions: Walking suggestions are randomized at a decision point only if a participant is available for treatment at that time. Participant is deemed unavailable (and is, thus, not randomized) under four conditions: (1) if the participant received another intervention (an anti-sedentary suggestion) within the last hour; (2) if the participant has walked more than 250 steps in the last hour; (3) if the participant has had over 2000 steps in the last two hours (to avoid annoying participants who have recently finished a prolonged bout of activity); and (4) if the participant’s phone cannot be reached (i.e., the participant is offline) and, thus, a notification cannot be sent.

#### 2.4.3. Anti-Sedentary Suggestions

Anti-sedentary suggestions are randomized using a personalization algorithm [24] developed by our team to manage user burden that can result from sending too many notifications. While some systems (e.g., Apple Watch, Fitbit) provide movement reminders whenever the user has been inactive for a certain period of time, for individuals with sedentary jobs or lifestyles this can lead to a large number of interventions each day, resulting in high user burden and potential intervention abandonment. HeartSteps attempts to deal with this problem by imposing an average budget on anti-sedentary messages. Specifically, HeartSteps aims to provide an average maximum of 2.5 anti-sedentary messages per day. To avoid situations where all anti-sedentary messages are sent close together (e.g., in the morning, in the case of individuals with office jobs), provision of anti-sedentary messages is handled by an algorithm that uses each participant’s data to make predictions about the amount and timing of sedentary time a person is likely to have each day. Based on these predictions, the algorithm then randomizes anti-sedentary messages to try to distribute them, within the budget limit, over the course of the whole day. Each participant’s sedentary status is assessed every five minutes when they are wearing the Fitbit with the HeartSteps clock face installed, and the algorithm runs whenever the participant has had at least 40 min of time with fewer than 150 steps. For an inactive individual, this means that this algorithm can run—and anti-sedentary messages are randomized—over a hundred times per day. As with walking suggestions, all randomization probabilities are saved for each participant to enable post-study analyses.

The proximal outcome for anti-sedentary messages is time to sedentary-behavior-disruption. We hypothesize that when individuals are sent an anti-sedentary message, they will get up and move (i.e., they will start accruing steps) sooner than when they have been inactive but are randomized not to receive an anti-sedentary message.

Availability for anti-sedentary messages: As with the walking suggestion, anti-sedentary suggestions are only randomized if a participant is available for this intervention. Participants are unavailable for anti-sedentary messages if (1) they are not wearing a Fitbit with a functioning HeartSteps clock face; (2) they have had more than 150 steps in the previous 40 min; (3) another intervention (a walking suggestion or another anti-sedentary suggestion) was provided in the last 60 min; (4) they had over 2000 steps in the previous two hours; and (5) the participant is offline and the phone cannot be reached to send a notification.

### 2.5. Measures

The HeartSteps II study involves both active (i.e., via self-report) and passive (i.e., sensor-based) assessments. Active assessments are conducted via RedCap-based questionnaires at baseline and 1-year follow-up (or when a participant withdraws), and via phone-based questionnaires over the course of the study. Passive measures are collected via the Fitbit activity tracker and through logging of participants’ interactions with the HeartSteps application (i.e., app page views).

#### 2.5.1. Baseline and Follow Up

The baseline and follow-up measures are shown in Table 1.

During the onboarding process, participants were emailed a RedCap link which directed them to a self-report questionnaire which collected demographic information including age, race, marital status, household size, employment status, and level of education. This questionnaire also gathered information on baseline measurements for psychosocial variables of interest and the participant’s amount of mobile phone usage. During the offboarding process, participants are emailed another RedCap link to complete an exit questionnaire which reassessed several of the psychosocial variables collected at baseline, as well as the effects of COVID-19 outbreak on the participant’s physical activity and HeartSteps application usability. These data will serve as baseline and follow-up measurements for longitudinal data analyses and modeling efforts.

#### 2.5.2. Ecological Momentary Assessment (EMA)

EMA measures and timing are detailed in Table 2. How they are collected is detailed in this section.

##### Daily Questionnaires

Daily questionnaires are part of the “morning survey” interaction that is sent to participants as a push notification at 6 a.m. each morning. If participants open the notification, they are shown the weather forecast for the day, a motivational message (if they are randomized to receive one), and a six-question questionnaire about their day (order of questions is randomized).

##### Weekly Questionnaires

Weekly questionnaires are provided as part of the weekly reflection, which is, by default, sent to participants at 8 p.m. on Sunday evenings. The questionnaires ask participants to assess the just-finished week and report any barriers they encountered to being active. Weekly reflection includes three instruments: weekly survey (8 questions in random order), barrier survey, and goal self-efficacy question. This question is presented right below the interface for adjusting the weekly goal and is intended to help participants set challenging but achievable goals.

##### Activity Questionnaires

Activity questionnaires are programmed to prompt participants with a brief survey after they have finished a bout of activity (i.e., when HeartSteps system receives a signal from the Fitbit API that a participant has a new activity record). These questionnaires are intended to elicit information about the context around the activities that participants perform. To minimize participant burden, during the study, activity questionnaires are sent with a low base probability of 0.1, but are sent for each detected activity (i.e., with probability of 1) during the EMA bursts described below.

##### EMA Bursts

EMA bursts are scheduled to occur, on average, every three months and they last for seven days. During this period participants are prompted with two types of instruments: (1) activity questionnaires and (2) walking-suggestion questionnaires. Walking suggestion questionnaires are intended to elicit information that would help better elucidate the contexts in which walking suggestions do and do not work.

#### 2.5.3. Passive Measures

The HeartSteps II studies uses two sources of passive measures: the Fitbit activity tracker and HeartSteps app use logs.

##### The Fitbit Activity Tracker

The Fitbit activity tracker is used to collect (1) minute-level step counts; (2) minute-level heart rate data; (3) activity bouts; and (4) GPS location information. Of these, activity bouts require some further discussion. Fitbit trackers contain algorithms to automatically detect 10-min or longer bouts of walking, cycling, running, swimming, and elliptical and StairMaster workouts. Furthermore, if the Fitbit detects that a person is being active but cannot determine what activity is being performed, it may record a bout of “sport”—its terminology for a generic activity. Each detected activity includes start and stop times (and, thus, duration), as well as associated minutes of MVPA. MVPA minutes (“active minutes” in Fitbit terminology) are calculated based on detected heart rate and are recorded only if the detected heart rate is Zone 2 or higher. Due to the need to conserve battery, however, Fitbit heart rate sensing in the 24/7 mode can be unreliable, so automatically detected active minutes may not always be accurate, especially for moderate-intensity activities.

In addition to automatically detecting activities, the Fitbit tracker can also be put into a workout mode, where participants manually mark on the watch the start and stop times for an activity. This allows participants to record activities that the watch cannot automatically detect (e.g., yoga). In the workout mode, the heart rate sensor samples heart rate at a higher frequency and, thus, resulting activity records may have more accurate active minutes.

##### HeartSteps Use Logs

HeartSteps use logs record time stamps for every page view in the HeartSteps app, including opening of notifications, opening the app, viewed pages within the app, and opening of surveys. The one piece of information that use logs are not able to capture is whether HeartSteps notifications (e.g., walking suggestions) were seen without being opened, such as when they automatically expand on the iOS lock screen. Since both Android and iOS record only when a notification is opened (tapped on), such non-interactive views cannot be logged.

## 3. Participants and Procedures

### 3.1. Recruitment

In response to the COVID-19 pandemic, recruitment was moved to a no-contact platform. Participants are being recruited through email threads, community newsletters, online forums, student organizations, flyers in LA county, and local media outreach.

### 3.2. Power Calculations

The study is powered at 60 participants. Allowing for a 25% dropout rate, we projected that we would need to recruit at least 80 individuals for the HeartSteps II study. Because this is a single-arm micro-randomized trial, the study was powered to detect effects for the three components that were micro-randomized: motivational messages (randomized daily), walking suggestions (randomized 5 times a day), and ant-sedentary suggestions (randomized whenever the participant has been sitting for 40 min or more. As the motivational messages are the least frequently randomized component, as well as a component with the least variation in when they are provided (i.e., as part of the morning survey at 6 a.m. every morning), we sized the study to be able to detect a very small (d = 0.05) gradually decreasing effect for this component. To detect this effect with 90% power and type II error rate of 0.05, the study requires 46 participants. Assuming a 20% attrition rate, which is higher than we have had in previous studies of this sort, the total needed sample size was 58 participants, which we rounded up to 60. With this participant number we are overpowered for detecting even very small effects for the other two micro-randomized components.

### 3.3. Eligibility

The study aims to enroll at least 80 participants between the ages of 18 and 65 years old that are classified as being overweight, sedentary, but otherwise healthy. We have cautiously calculated an expected attrition of 25%, and thus aim for a final sample of 60. The inclusion criteria include participants who have a Body Mass Index (BMI, weight in kilograms divided by height in meters squared) between 25–45, have a sedentary lifestyle, have the ability to participate in mild or moderate physical activity, live in Southern California, and are competent to give informed consent. The participants must own either an iPhone with iOS 8 or above or an Android phone with Version 7 or above to be able to install the HeartSteps app and Fitbit app. Participants must also be willing to follow study protocol, including regularly carrying a mobile phone, using the HeartSteps application, answering phone-based questionnaires, and wearing the Fitbit Versa activity tracker at least 8 h a day. Participants must also be fluent in English because HeartSteps is only programmed in English. Candidates will not be eligible if they are incapable of giving informed consent, or if they have a psychiatric disorder that limits their ability to follow study protocol, including psychosis and dementia.

To assess eligibility, a remote screening process is being used. The process includes assessments of: (1) body mass index (BMI) based on the candidate’s self-reported height and weight; and (2) physical activity, measured by the IPAQ questionnaire. If the candidate engages in vigorous activity that spans at least three days and leads to a total of at least 1500 MET min or 7 or more days of any combination of exercises that exceeds a total of 3000 MET min, then it is determined that the participant engages in physical activity that exceeds a moderate level, making the participant ineligible for the study. Table 3 shows HeartSteps inclusion and exclusion criteria.

### 3.4. Initial Screening, Orientation, and Run-in Procedures

In response to the COVID-19 pandemic, all participant interactions are being done remotely. All potential participants are referred to the project specialist, who sends them a link to complete the screening questionnaire and IPAQ through RedCAP. Eligible individuals are sent informed consent forms and detailed study information. After participants return a signed consent form, they are sent a Fitbit Versa smartwatch and a $20 gift card, to thank them for completing the baseline measurements on RedCAP. Once the Fitbit is delivered, participants are sent an email with instructions on how to set up both their Fitbit and cell phone for the study. These instructions also include the enrollment code which participants will use to log into HeartSteps.

For the first week of the study all participants are instructed to wear their Fitbit for at least 8 h every day within the hours of 6 a.m. to 10 p.m. in order to collect baseline activity measures. The HeartSteps app remains locked until they have completed 7 days of Fitbit wear. Should participants fail to meet these criteria, they will not be invited to continue the study and are asked to return their equipment.

Once the full baseline week of wear is completed, the project specialist sets up a phone or video check-in with the participants to complete the final onboarding step. During the call, the participant is walked through how to use the HeartSteps app (e.g., setting preferred walking suggestion times) and the HeartSteps clock face. The project specialist also makes sure that the participant is able to receive HeartSteps notifications and that these show up on the participant’s Fitbit. Study procedures are reiterated, and at the end of the call, the project specialist emails the participant a welcome packet with user manuals and a Fitbit best practices document.

### 3.5. Participants

**Figure 3:**
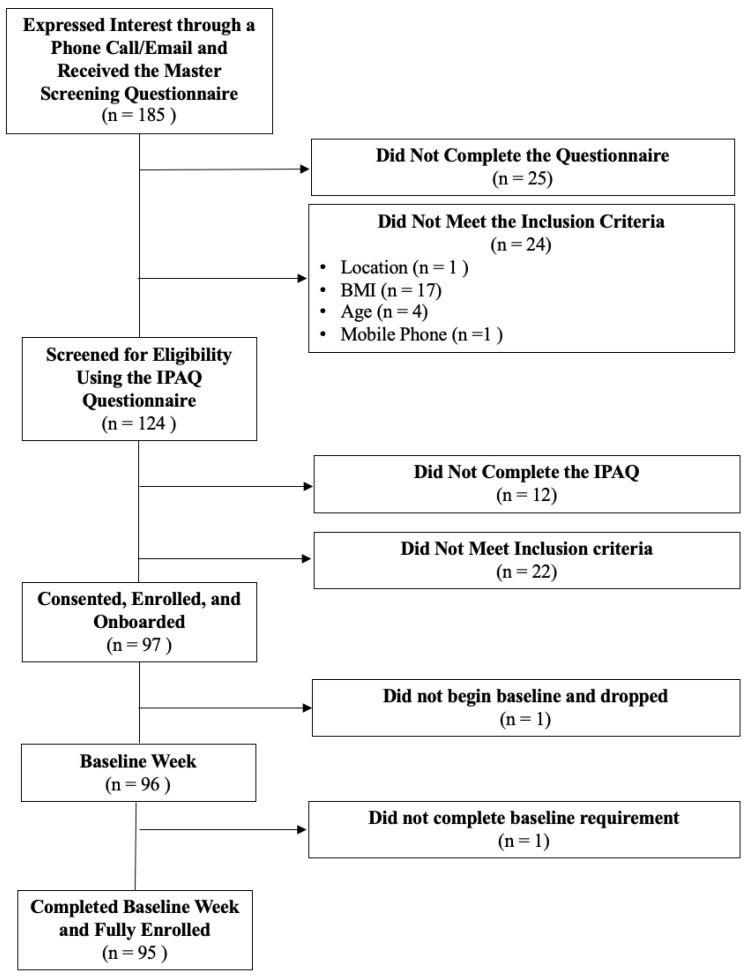
HeartSteps II consort diagram.

Because some participants expressed the desire to come in to the study with family members, we recruited extra participants to aim for 60 independent participants at the end of the one-year study. At the close of recruitment, we had 95 fully enrolled participants. The consort diagram is show below in Figure 3.

The sample characteristics are included in Table 4, below.

### 3.6. Treatment Fidelity (Monitoring, Contacting Protocol, Adherence)

#### 3.6.1. Monitoring Data Collection

Study adherence is monitored both automatically through the HeartSteps server and manually by the HeartSteps team through the system’s researcher dashboard. Several types of participant compliance are monitored, each with a distinct follow-up protocol.

##### No Fitbit Data

If a participant has no Fitbit Data recorded within 48 h, an automated adherence text message is sent to notify the participant that their Fitbit has not synced with HeartSteps in a few days. If the participant still has no Fitbit Data recorded after 72 h, then the same automated text message will be sent. When no steps are recorded for one week, a team member will call the participant to notify them of the syncing problem.

##### No Interaction with the HeartSteps Application

If the participant has not interacted with the HeartSteps application data for 96 h an automatic text message is sent. Participants are also manually monitored through the Dashboard using the “Last Page View” feature. Those who have been inactive on the application for over a week will receive a phone call from one of the HeartSteps team members.

##### Morning Survey Non-Compliance

Morning Check-In completion is monitored using the “Morning Messages” tab on the Dashboard. Displayed in this section is the last 7 days of morning surveys sent to each participant and whether they completed the survey. If it is found that the participant has not completed the morning survey three days consecutively, a reminder message is sent by a HeartSteps team member. After 5 days of non-compliance, the participant will receive the same reminder message. After a week of continuous non-adherence, the participant will receive a phone call from a HeartSteps team member.

### 3.7. Burst Adherence Protocol

Due to the nature of the EMA Bursts, a more rigorous adherence protocol is utilized in order to ensure survey completion. A day before the start of the EMA Burst week, participants will be a message as a reminder.

If the participant has completed 0 activity surveys, 0 walking suggestion surveys and/or has not worn their watch in 24 h, they will receive either this message: “It looks like you haven’t completed any of the burst surveys recently. Please try your best to complete these additional surveys as they are an important part of the study,” or this message “It looks like you haven’t worn your watch recently. Please try your best to wear your watch as it is an important part of the burst week” depending upon their specific non-adherence. Both messages are accompanied by contact information of study personnel should the participant be experiencing any technical difficulties.

In the event that the participant does not complete any of the EMA surveys and/or does not wear their watch for 48 h, they will receive a phone call from a HeartSteps team member.

### 3.8. Data Storage, Security and Privacy

As it was initially used for a study conducted at Kaiser Permanente Washington Health Research Institute, HeartSteps infrastructure was designed to comply with the security and privacy requirements of a major health system. Google Cloud platform that hosts the server part of the system is built on the infrastructure that is fully HIPAA-compliant, the HeartSteps mobile application is sandboxed on both iOS and Android, and all communication between the mobile application and the HeartSteps server is encrypted using SSL, the same technology used to encrypt communication with financial and medical institutions.

## 4. Modeling and Data Analysis

### 4.1. Analyses of Micro-Randomized Intervention Data

Since the HeartSteps II study is an MRT, the primary analyses for the micro-randomized intervention components will follow standard MRT data-analytic approaches [11]. Specifically, for each micro-randomized component—walking suggestions, anti-sedentary suggestions, and morning motivational messages—the primary analyses will assess its average, across-time, effect on its proximal outcome, and the change in this effect over time. Secondary analyses will examine the time-varying moderation of the components’ effects, such as moderation by dose (number of recently sent messages), weather, or current activity level. All analyses will be conducted using the centered and weighted least square method [35], a generalization of linear regression that enables estimation of causal treatment effects with robust inclusion of covariates to reduce noise. Like GEE [36] or multi-level models [37], this approach takes account of the nested nature of the MRT data (decision points nested within participants) and the resulting within-person correlation of the outcome across time.

### 4.2. Modeling Framework Development

Future ability to optimize interventions—i.e., to determine the best times and types of intervention to provide—requires the ability to predict behavioral response to various interventions in different contexts over time. These predictions can be made using computational behavioral models that estimate individuals’ responses to different interventions across time and contexts. This project aims both to build specific dynamic computational models of intervention response and engagement using the newly collected data, as well as to develop generalizable modeling approaches and tools that enable behavioral scientists to express a broad range of dynamic hypotheses about processes that shape heath behavior and health behavior change. Thus, the current study was designed, in part, to collect a diverse set of data that can be used to develop and validate such modeling approaches. As we described in Section 2, the HeartSteps II trial combines the collection of temporally dense, sensed physical activity measures with self-reported measures including surveys and ecological momentary assessments. HeartSteps participants also produce an array of digital traces through interacting with the HeartSteps mobile application and intervention components. Although these data sources are very rich, they are also highly heterogeneous in terms of content, measurement frequency, and patterns of missing data. We also expect significant heterogeneity across participants in terms of the dynamics of the underlying health behaviors and related constructs, their response to intervention components, and their engagement with the study data collection process.

We are currently pursuing two approaches to modeling that can take advantage of—and account for—such varied data and the associated levels of missingness and sparsity: dynamic Bayesian networks, [38,39] and dynamical systems modeling [40]. In addition, we are developing guidelines for representing dynamic hypotheses about behavioral processes, including through the use of graphical representations that can be further operationalized into dynamic computational models.

#### 4.2.1. Computational Modeling with Dynamic Bayesian Networks

A dynamic Bayesian network (DBN) model [38,39] is a probabilistic model defined over a multivariate time series whose structure is specified by an associated directed graph. We say that one random variable is the parent of another random variable in the DBN if there is a directed edge in the DBN graph the one to the other. The DBN model specifies a probability distribution over all random variables as a product over a set of factors that are each local to a single random variable. In particular, we must specify a probability distribution over each random variable conditioned on its parents. This is accomplished by selecting a probability distribution for each random variable and specifying parameterized equations relating the parameters of the distribution to the values of the parent variables. The unknown parameters of these distributions are then estimated from data.

For our purposes, a key advantage of DBNs is that they are an extremely flexible modeling class that can be used to model processes with constructs assessed on different time scales and that include different patterns of missingness. To make modeling using DBNs more accessible, our current work includes the development of a domain-specific probabilistic modeling language that facilities the specification of DBN models as well as the application of these models in both the simulation and inference settings. Along with guidelines for how to represent dynamic hypotheses graphically, our hope is that this tool will provide much needed support for theorizing about the dynamics of health behaviors by enabling rapid model refinement and model-based exploratory data analysis.

#### 4.2.2. Modeling with Continuous Dynamical Systems

Our second thread of modeling work builds on our prior work in dynamical systems modeling [41,42,43,44,45]. Applied in engineering to model systems such as electrical circuits, mechanical systems, epidemiological processes, chemical plants, etc., dynamical systems modeling relies on physical (or similar) principles—e.g., Newton’s Laws, or conservation of mass—to represent how systems evolve over time. In the domain of human behavior our team found it convenient to use the fluid analogy (a set of tanks and valves) to specify the flow of influences in a system and how those influences combine to affect an outcome of interest. A fluid diagram that specifies a dynamic hypothesis about a process is operationalized mathematically through a set of differential or difference equations arising from the principle of conservation of mass applied to latent variables. The resulting model is used to represent the deterministic evolution of the underlying system. Any uncertainty is assumed to be additive random noise in a similar manner to ordinary regression. This approach is less general in some ways than the DBN framework, but it does not require specification of probability distributions over variables. Well-established computational approaches can, therefore, be used to estimate the free parameters of the model using maximum likelihood approaches.

An advantage of dynamical systems models is that they are fundamentally idiographic. They are, in other words, intended to represent the behavior of a single unit (in this case person). Dynamical systems models are thus particularly well suited for investigating how psychosocial and contextual factors that are most influential on a person’s health behavior vary from one individual to another.

In the current project, we are extending our prior dynamical-systems modeling work in two ways: first, we are exploring the use of the Model on Demand (MoD) estimation framework, which allows estimating parameters of a low-order parametric model based on the data from a local regressor temporal neighborhood that changes “on demand” based on user-specified statistical criteria [46,47]. This approach allows flexibility in the modeling framework to capture nonlinearity, without having to specify a single “global” model. It represents an appealing approach to addressing the bias-variance tradeoffs that are inherent to all estimation problems. The second extension is the use of discrete Simultaneous Perturbation Stochastic Approximation (DSPSA) [48] as a search procedure for determining model features and MoD design parameters in an efficient manner. DSPSA mimics gradient descent, iterating towards a solution by approximating a gradient from a simultaneous two-sided perturbation of the features and parameters. As such, the method does not require a closed-form objective function, which are often unavailable or difficult to specify. DSPSA is simple to implement and efficient in distilling optimized models from large volumes of data.

## 5. Discussion and Conclusions

There is growing realization in the behavioral science community that our traditional theories of health behavior and health behavior change do not adequately capture the dynamic nature of these phenomena and the full range of factors that influence them. The advances in mobile and wearable technology have made it possible to capture both a broader range of factors that may influence health behaviors and at a much higher granularity—enabling sampling of certain constructs and behaviors many times per day. Yet, while it clearly presents important opportunities, it has been unclear how this new data can be used to develop better, more dynamic theories of the processes that shape health behaviors and health behavior change.

The parent project of this study aims to address that question by developing modeling frameworks that can be used to mathematically specify a set of dynamic hypotheses about how psychosocial, contextual, and environmental factors affect a health behavior and how those influences unfold and change over time. The current study supports this work by collecting rich, longitudinal data that can be used both as a testbed for postulating and testing such dynamic hypotheses, as well as a concrete example of the characteristics of the data—timescales, granularity, missingness patterns, etc.—that the modeling frameworks that we are developing will need to be able to handle. The modeling proposed in this paper that uses fluid analogies and Bayesian modeling to describe behavioral theories and interventions has not been done before, to the best of our knowledge. Publications referenced in the document provide details that are meaningful to both the novice and the expert, the behavioral scientist and the engineer/computer scientist [40,41,42,43,44,45].

The analyses of the MRT data proposed here will provide evidence that can be used to inform decision rules for exactly when and how intervention components should be deployed through highly personalized, data-informed timing. This approach is hypothesized to optimize the intervention as well as reduce habituation. If successful, therefore, this study will contribute both new knowledge about the specific interventions contained in the HeartSteps II system and the patterns of engagement with mHealth interventions, as well as templates for how to build a new class of behavioral theories that, hopefully, more accurately reflect the richness and complexity of human health behavior.

## Figures and Tables

**Table 1:** Baseline and Follow-up Measures.

Measures	Answer Categories (Number of Items)
Demographic Information *	Age, Race, Marital Status, Household Members, Employment Status, Level of Education (6 items)
Autonomy to Decide Schedule *	Strongly Disagree to Strongly agree (3 items) (5-point Likert)
Mobile Phone Usage *	More than 30 times per day; Between 10 and 30 times per day; Between 5 and 9 times per day; 3 or 4 times per day; 1 or 2 per day; Less frequently than once per day (1 item)
Ten-Item Personality Inventory (TIPI) [25] *	Disagree strongly to Agree Strongly (10 items) (7-point Likert scale)
Stress (Perceived Stress Scale—PSS) [26] ^	Never to very often (5 items) (5-point Likert scale)
Routine *	Definitely a morning type; Rather more a morning type than an evening type; Rather more an evening-type than a morning-type; Definitely an evening type (1 item)
Self-efficacy for Physical Activity [27] ^	Not at all confident to extremely confident (5 items) (5-point Likert)
Motivation for Physical Activity [28] ^	Not true for me to very true for me (19 items) (5-point Likert)
Value of Physical Activity [29] *	Extremely worthless to Extremely valuable (1 item) (5-point Likert)
Intention in Physical Activity [30] *	Not at all to very much (1 item) (5-point Likert)
Perceived Effects of Physical Activity ^	How long does it take before you notice the positive (3 items)/negative (3 items) effects of physical activity on; emotional well-being, physical heath, mood
Social Support in Physical Activity ^	Never true, sometimes true, always true, not applicable (6 items)
Neighborhood Walkability [31] *	Strongly disagree to strongly agree (6 items) (4-point Likert)
Social Isolation Index [32] *	Rarely or never, once a month, several times a month, at least once a week (6 items)
Physical Activity During COVID-19 Outbreak [33] +	Yes, no, decline to answer (3 items)
App Usability (Mobile App Rating Scale (MARS) [34] +	Item specific answer categories (20 items)

* denotes measures collected on baseline only; + denotes measures collected on follow-up questionnaire only. ^ denotes measures collected on both baseline and follow-up questionnaires.

**Table 2:** Ecological Momentary Assessments and Timing.

Morning Questionnaire (Once Daily, Available after 6:00 a.m.)				
Item	Answer Categories	* Current	Pro-Spective	Retro-Spective
How well rested do you feel this morning?	Not at all—Very (5-pt. Likert)	X		
How busy is your day going to be today?	Not at all—Very (5-pt. Likert)		X	
If I walk or exercise today, it’s because it’s important to my life	Not at all true—Very true (5-pt. Likert)		X	
If I walk or exercise today, it’s because other people think I should	Not at all true—Very true (5-pt. Likert)		X	
How committed do you feel this morning in being physically active today	Not at all—Very (5-pt. Likert)	X		
Which of these best describes how you feel this morning	(Choose one) sad, energetic, stressed, relaxed, fatigued, happy, tense	X		
**Weekly questionnaire (once a week, available Sunday)**				
People in my life supported me in my efforts to be more active this past week (not at all true to very true	Not at all true—Very true (5-pt. Likert)			X
How much are you enjoying physical activities you did this week?	Not at all—Very much (5-pt. Likert)			X
Over the past week, how socially connected you felt?	Not at all—Extremely (5-pt. Likert)			X
This week, I walked or exercised because I felt restless, stressed, or in a bad mood	Not at all true—Very true (5-pt. Likert)			X
How well is physical activity currently fitting into your daily routine	Not at all—Very much (5-pt. Likert)			X
This past week, I walked or exercised because other people think I should	Not at all true—Very true (5-pt. Likert)			X
This past week, I walked or exercised because it’s important for my life	Not at all—Very much (5-pt. Likert)			X
Over the past week, how lonely have you felt?	Not at all—Extremely (5-pt. Likert)			X
Did any of the following make it difficult for you to be active this week? (Barriers)	(Check all that apply) illness or injury; poor weather; sore muscles; no time/too busy; no place to be active; personal safety; traffic safety; travel, open response category			X
Is this barrier likely to continue into next week?	(Choose one) Yes, No, I don’t know		X	
How confident are you that you’ll reach your goal	Not at all—Very much (5-pt. Likert)	X	X	
**Activity Questionnaires (outside of burst weeks, probability of 0.1 after activity is detected)**				
How much did you enjoy the activity you just did?	Not at all—Very much (5-pt. Likert)			X
How well did this activity fit into your day?	Not at all—Very much (5-pt. Likert)			X
I did this activity …	(Choose one) alone, with friends, with family, with colleagues, with someone else			X
I did this activity partly because I wanted to be more active	Not at all true—Very true (5-pt. Likert)			X
I did this activity because other people thought I should	Not at all true—Very true (5-pt. Likert)			X
**EMA Bursts—once every three months for seven days**				
Activity questionnaires (see above) after completion of each detected activity				
Walking suggestion questionnaires (see below) five times per day				
How busy are you right now?	Not at all—Very (5-pt. Likert)	X		
Right now, how relaxed do you feel?	Not at all—Very (5-pt. Likert)	X		
Right now, how tense do you feel?	Not at all—Very (5-pt. Likert)	X		
Right now, how energetic do you feel?	Not at all—Very (5-pt. Likert)	X		
Right now, how fatigued do you feel?	Not at all—Very (5-pt. Likert)	X		
Right now, how happy do you feel?	Not at all—Very (5-pt. Likert)	X		
Right now, how sad do you feel?	Not at all—Very (5-pt. Likert)	X		
Right now, how stressed do you feel?	Not at all—Very (5-pt. Likert)	X		
How committed do you feel right now to being physically active today?	Not at all—Very (5-pt. Likert)	X		
Given what’s going on right now, I will be able to be active in the next hour.	Strongly disagree—Strongly agree (5-pt. Likert)		X	

* Current items ask about right now, for instance asking about your mood right now. Prospective items ask about the future, for instance asking how busy your day is going to be. Retrospective questions ask about the past, for instance asking about any barriers you experienced to physical activity in the last week.

**Table 3:** HeartSteps Inclusion and Exclusion Criteria.

Inclusion Criteria
BMI between 25–45 kg/m^2^
18–65 years of age
Competent to give informed consent
Own either an iPhone with iOS 6 or above or an Android phone with Version 7 or above.
Willing to participate in the study protocols including
Regularly carrying a mobile phone
Using the HeartSteps application using the HeartSteps application,
Answering phone-based questionnaires
Wear the Fitbit Versa activity tracker at least 8 h a day.
Fluent in English
Residing in Southern California
**Exclusion Criteria**
Mentally incapable of giving informed consent,
Psychiatric disorder that limits the patients’ ability to follow study protocol, including psychosis and dementia.
Non-English speaking
Orthopedic problems that prevent participation in a walking program
Significant peripheral neuropathy
Vigorous Activity that spans at least three days and leads to a total of at least 1500 MET min *or* 7 or more days of any combo of exercises that exceeds a total of 3000 MET min (Based on IPAQ scoring)

**Table 4:** HeartSteps II participant characteristics.

Demographics	Participants:
Gender	
Male	23
Female	72
Age	
18–20	2
21–34	37
35–44	25
45–65	31
Race/Ethnicity	
American Indian/Alaska Native	2
Asian	19
Black or African American	4
White	48
Middle Eastern	2
Hispanic/Latinx	39
More Than One Race	5
Other	10
Prefer not to respond	5
Level of education	
Some High school	1
High school graduate/diploma/GED	5
Some College or 2 year Degree	27
Bachelor’s degree (BS/BA/AB)	31
Some Graduate School	5
Graduate degree	25

## Data Availability

The data presented in this study will be available on request from the corresponding author after the completion of the study and main outcome articles have been published. The data are not yet publicly available due to the fact that the study is not yet complete.
